# Supplementary material for: Intracellular growth of Chlamydia trachomatis leads to global histone hypermethylation by impairing demethylation
Source: eLife. 2026 Sep 14;15:e110111. doi: 10.7554/eLife.110111 (PMC13668117; doi:10.7554/eLife.110111)
Supplement: Supplementary file 1 [file elife-110111-fig2-figsupp1-data1.pdf]

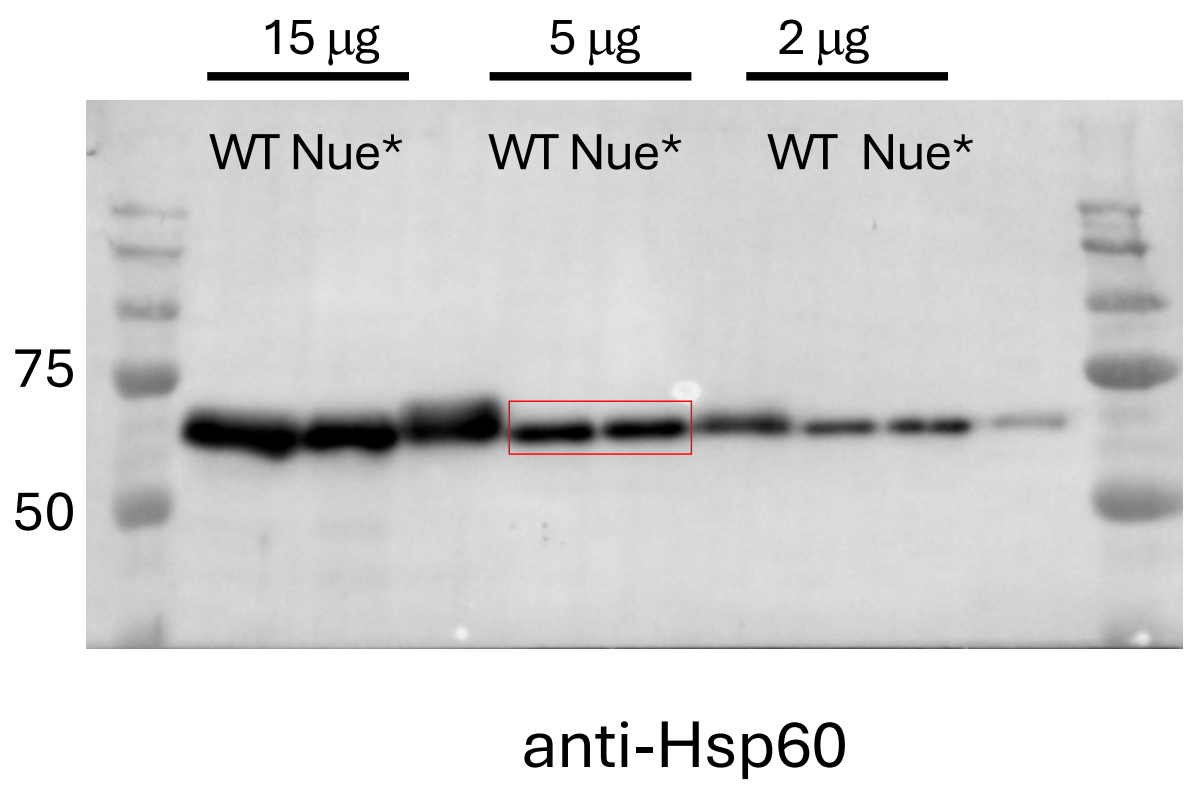

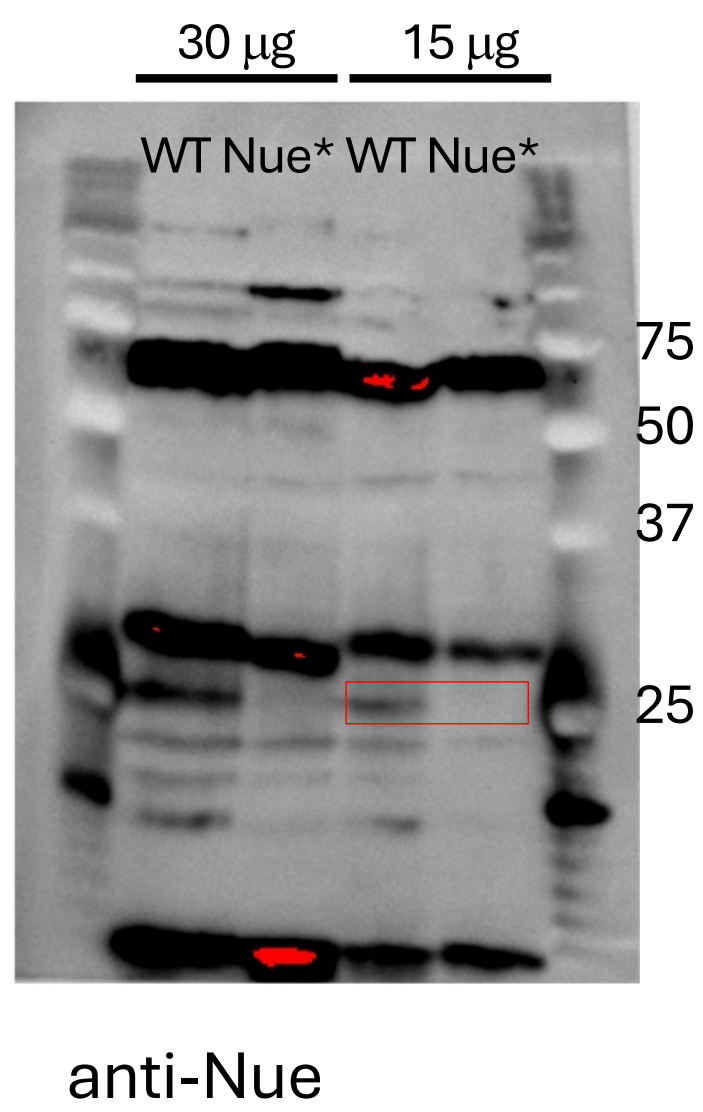

Figure 2_ figure supplement 1, Source Data 1. Original membranes
The indicated total amount of protein was loaded. Precision plus All-blue molecular weight markers were employed. The ROIs used for the figure are shown in red.
