# Supplementary material for: Intracellular growth of Chlamydia trachomatis leads to global histone hypermethylation by impairing demethylation
Source: eLife. 2026 Sep 14;15:e110111. doi: 10.7554/eLife.110111 (PMC13668117; doi:10.7554/eLife.110111)
Supplement: Supplementary file 2 [file elife-110111-fig6-figsupp1-data1.pdf]

Experiment 1

Filename: 2022-05-03-02.HSD5000

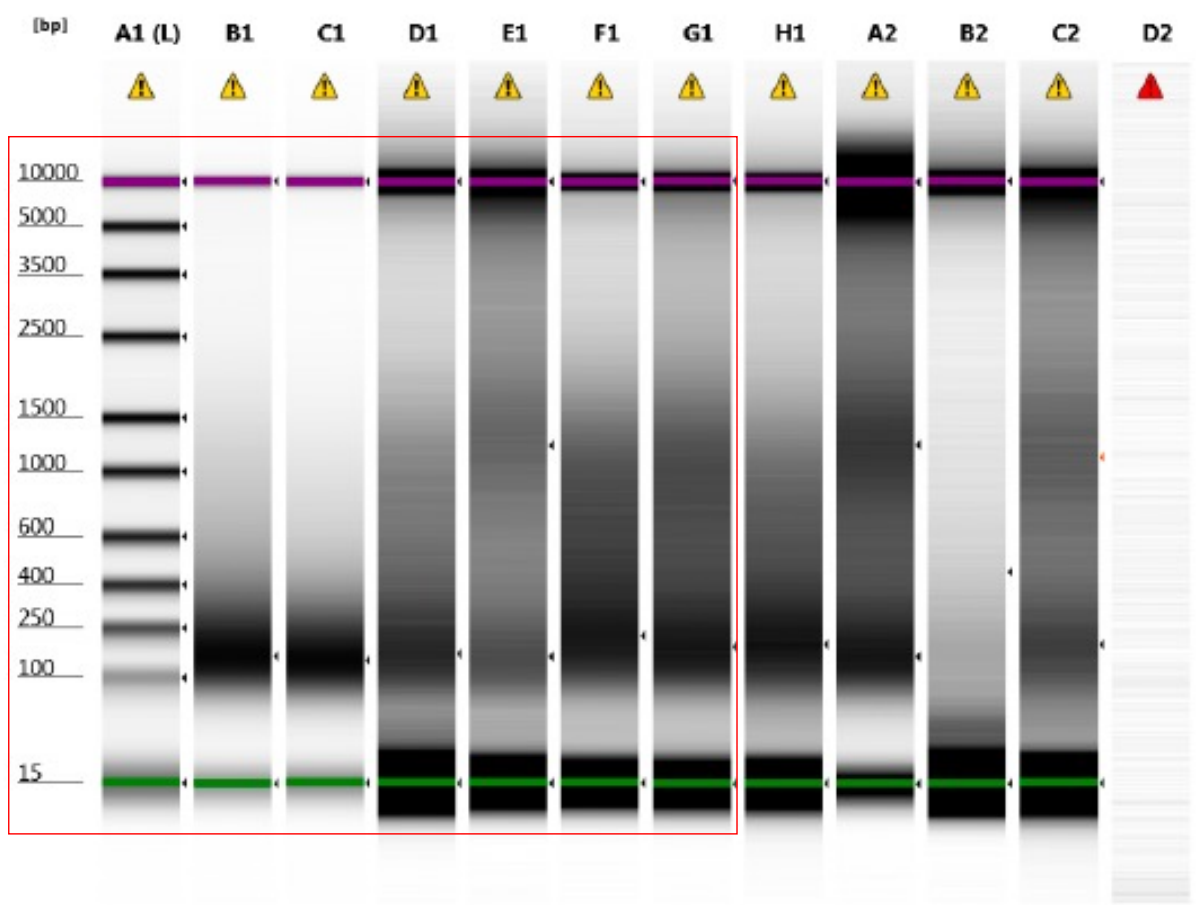

Default image (Contrast 50%), Image is Scaled to Sample

Sample Info

Well	Conc. [pg/µl]	Sample Description	Alert	Observations
A1	2260	Ladder	⚠	Caution! Expired ScreenTape device; Ladder
B1	3970	Input Cl-	⚠	Caution! Expired ScreenTape device
C1	2950	Input LGV	⚠	Caution! Expired ScreenTape device
D1	292	K4me3 Cl-	⚠	Caution! Expired ScreenTape device
E1	525	K4me3 LGV	⚠	Caution! Expired ScreenTape device
F1	863	K9me3 Cl-	⚠	Caution! Expired ScreenTape device
G1	933	K9me3 LGV	⚠	Caution! Expired ScreenTape device

Experiment 2

Filename: 2023-05-16-01.HSD5000

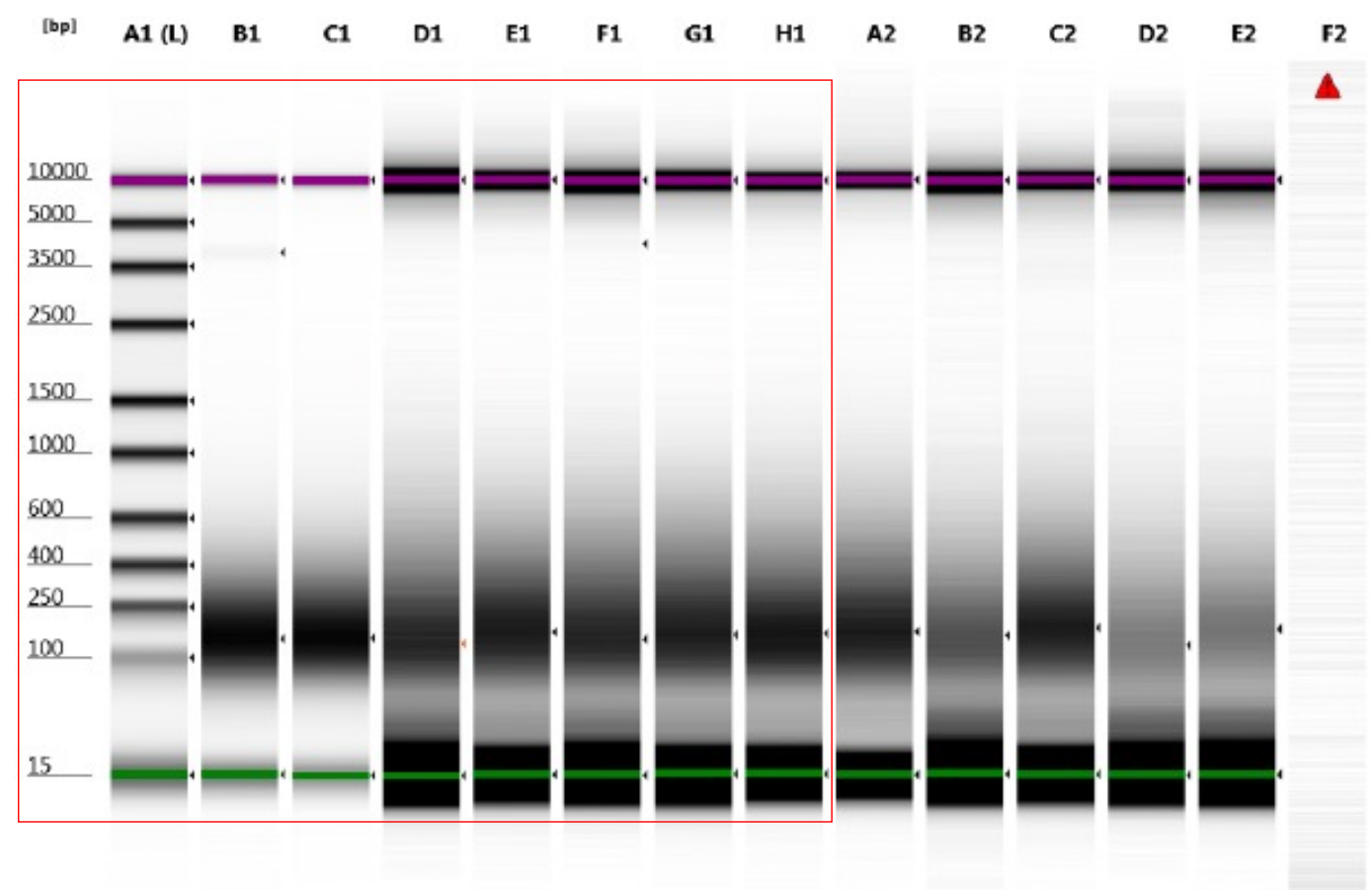

Default image (Contrast 50%), Image is Scaled to Sample

Sample Info

Well	Conc. [pg/µl]	Sample Description	Alert
A1	2240	Ladder	
B1	2300	Input cl-	
C1	2640	Input LGV	
D1	171	K4 cl-	
E1	278	K4 LGV	
F1	198	K9 Cl-	
G1	252	K9 LGV	

Figure 6_ figure supplement 1. Original gels. The ROIs used for the figure are shown in red.
