# Supplementary material for: Intracellular growth of Chlamydia trachomatis leads to global histone hypermethylation by impairing demethylation
Source: eLife. 2026 Sep 14;15:e110111. doi: 10.7554/eLife.110111 (PMC13668117; doi:10.7554/eLife.110111)
Supplement: Supplementary file 3 [file elife-110111-fig7-data1.pdf]

tRNA-Gly-GCC

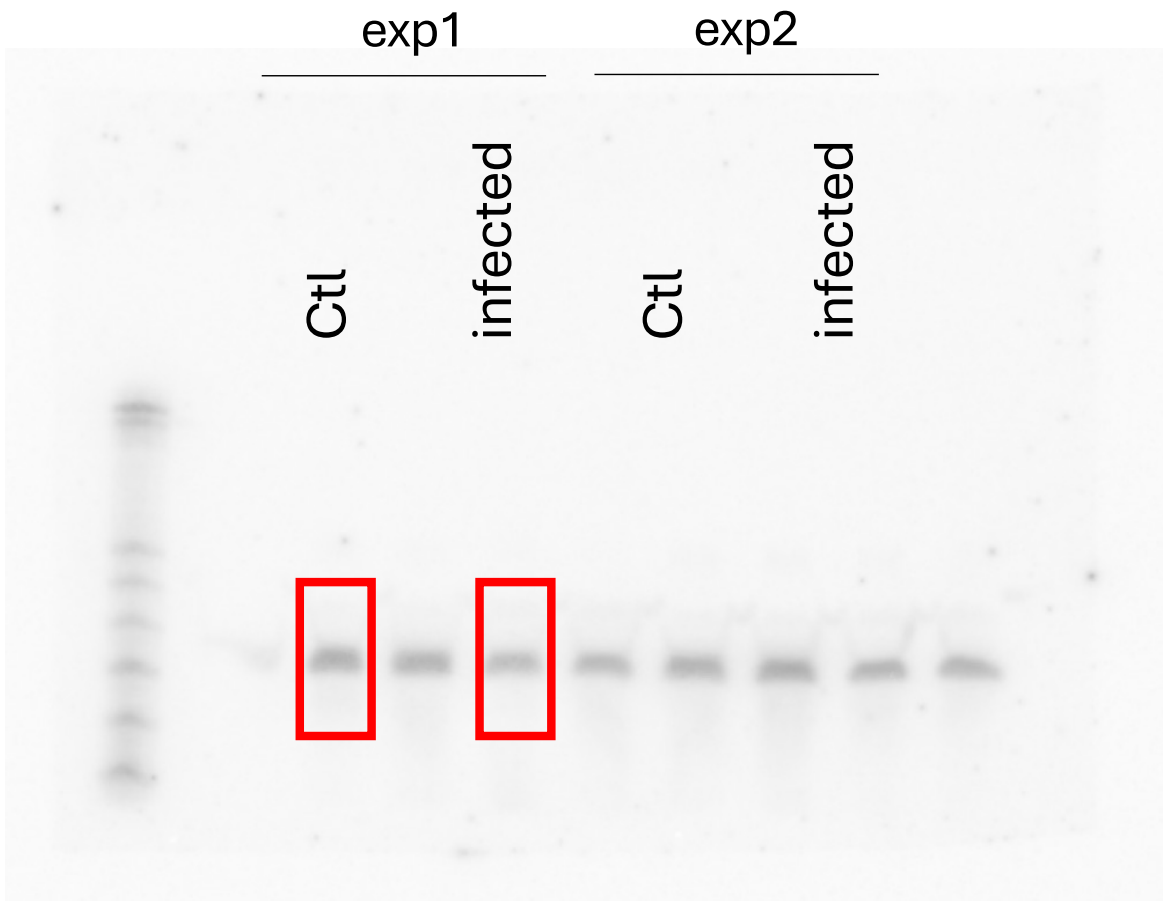

tRNA-Arg-UCG

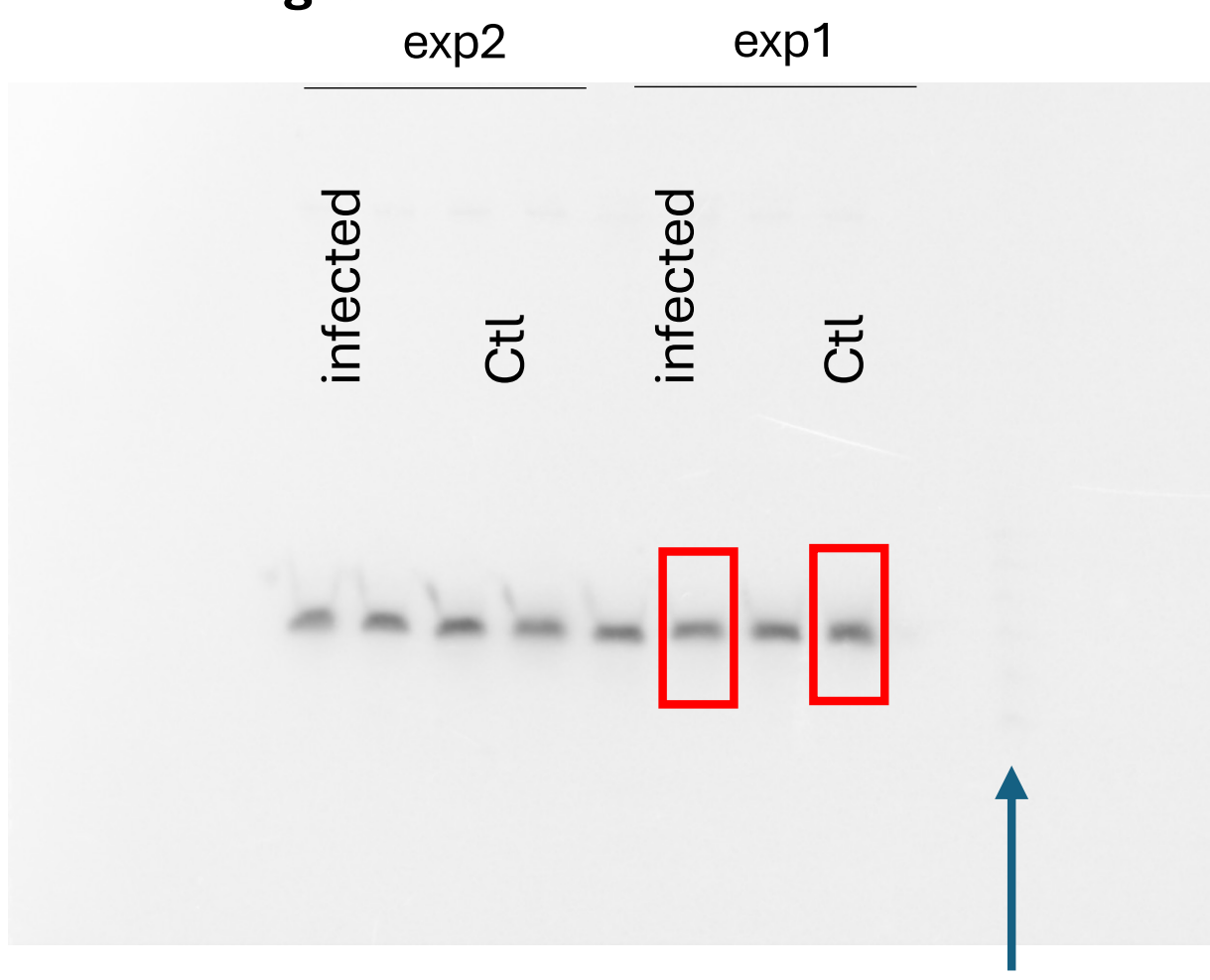

Note that the first lane is on the right, the image needs to be flipped.

U6 snRNA

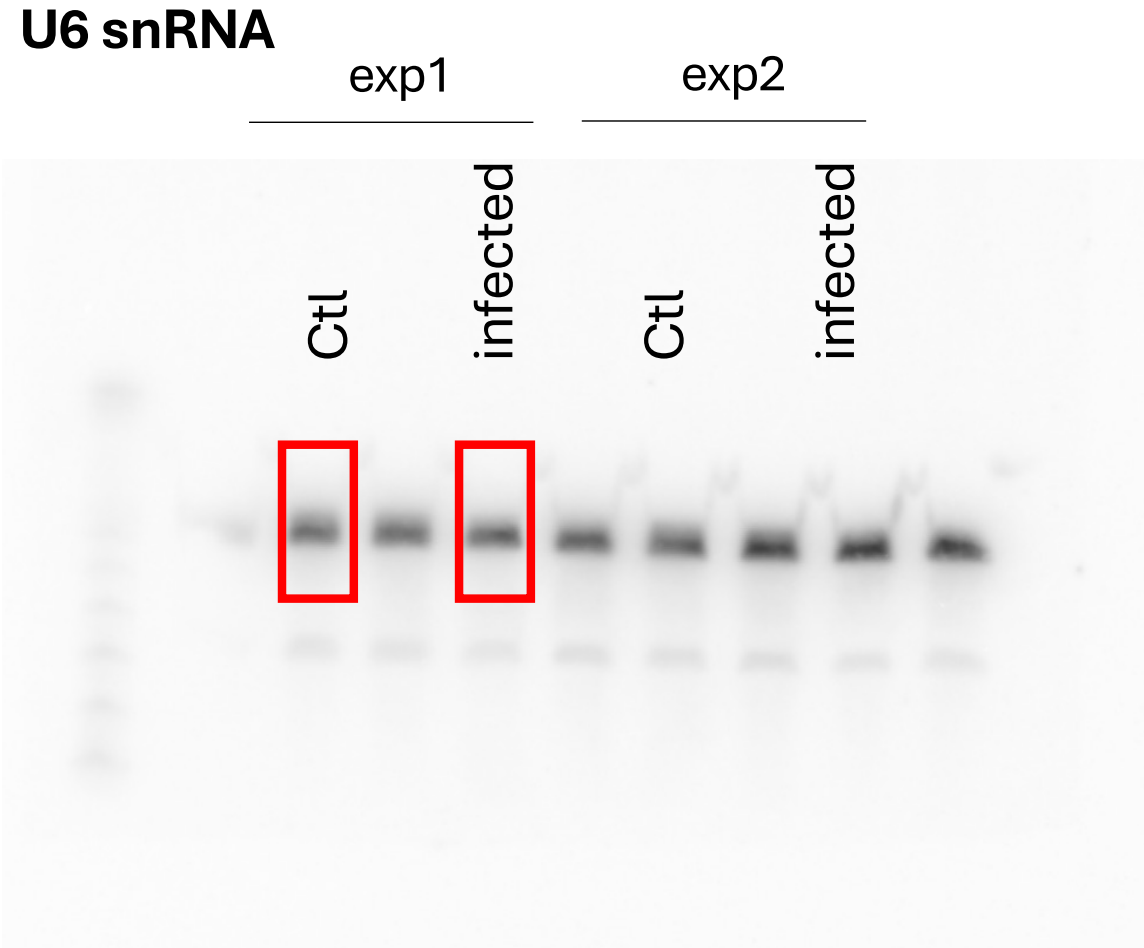

The blot was done on the tRNA-Gly membrane: the lower intensity bands in the gel corresponds to left-over signal from the tRNA-Gly blotting.

5S rRNA

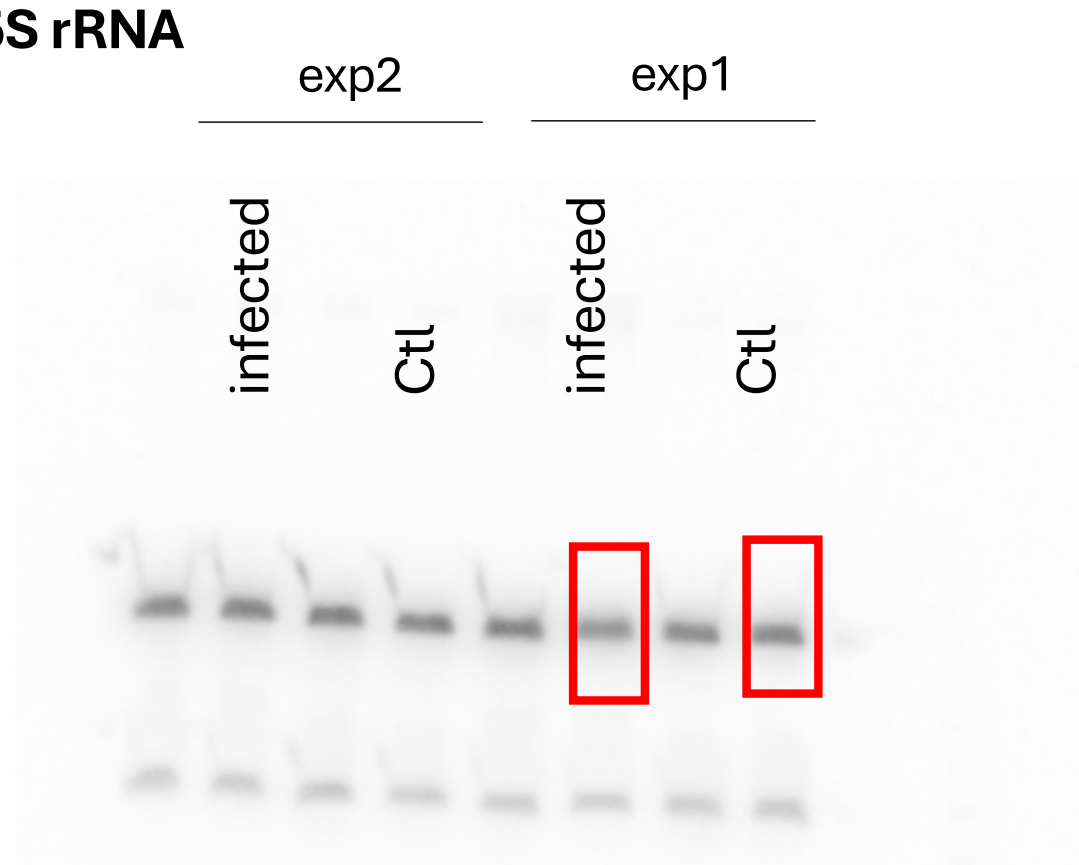

The blot was done on the tRNA-Arg membrane, the first lane is also on the right and the image needs to be flipped. The bands with lower intensity in the gel are the left-over signal from the tRNA Arg blotting.

Figure 7, Source Data 1. Original membranes corresponding to Figure 7 panel E. Two different experiments were loaded on these gels, the figure used samples from experiment 1. Non-labeled lanes correspond to irrelevant culture conditions. Decade™ Markers System (to make radiolabeled RNA markers; Thermo Fisher Scientific, Cat Nº AM7778) molecular weight markers were employed. The ROIs used for the figure are shown in red.
