# Supplementary material for: Intracellular growth of Chlamydia trachomatis leads to global histone hypermethylation by impairing demethylation
Source: eLife. 2026 Sep 14;15:e110111. doi: 10.7554/eLife.110111 (PMC13668117; doi:10.7554/eLife.110111)
Supplement: Supplementary file 5 [file elife-110111-supp6.docx]

**Supplementary File 6.** Antibodies used

|  | Target | Species | Supplier | Catalog # |
| --- | --- | --- | --- | --- |
| Primary  Antibodies | H3K9me3 | Rabbit | Abcam | ab8898 |
|  | H4K20me3 | Mouse | Abcam | ab78517 |
|  | H3K27me3 | Mouse | Bp biosciences | 25243 |
|  | H3K4me3 | Rabbit | Diagenode | C15410003 |
|  | H3K79me2 | Rabbit | Abcam | ab3594 |
|  | HA | Rat | Roche Diagnostics | Clone 3F10  #1 867 423 |
|  | Nue | Rabbit | Subtil lab | Pennni et al 2010 |
|  | Hsp60 | Rabbit | Subtil lab |  |
| Secondary  Antibodies | Alexa Fluor TM 488 goat anti-rabbit IgG (H+L) | Goat | Invitrogen | A-11034 |
|  | Alexa Fluor TM 647 goat anti-rabbit IgG (H+L) | Goat | Invitrogen | **A-21244** |
|  | Alexa Fluor TM 488 goat anti-mouse IgG (H+L) | Goat | Invitrogen | A-11029 |
|  | Alexa Fluor TM 647 goat anti-mouse IgG (H+L) | Goat | Invitrogen | A-21235 |
|  | Alexa Fluor TM 488 goat anti-rat IgG (H+L) | Goat | Invitrogen | A-11006 |
