# Supplementary material for: Intracellular growth of Chlamydia trachomatis leads to global histone hypermethylation by impairing demethylation
Source: eLife. 2026 Sep 14;15:e110111. doi: 10.7554/eLife.110111 (PMC13668117; doi:10.7554/eLife.110111)
Supplement: Peer review history

# Editor report: Editor's evaluation

**DOI:** 10.7554/eLife.110111.sa0

**Authors:** Jerry L Workman

**Affiliations:** https://ror.org/04bgfm609 Stowers Institute for Medical Research United States

This is a valuable study of changes in host genome histone methylation and transcription changes associated with Chlamydia infection. The data presented are solid and collectively support the model that histone hypermethylation is due to inhibition of demethylases. The findings provide insight into how pathogen-induced metabolic changes shape host epigenetic and transcriptional responses.

# Decision letter

**DOI:** 10.7554/eLife.110111.sa1

**Affiliations:** https://ror.org/04bgfm609 Stowers Institute for Medical Research United States

Our editorial process produces two outputs: i) public reviews designed to be posted alongside the preprint for the benefit of readers; ii) feedback on the manuscript for the authors, including requests for revisions, shown below. We also include an acceptance summary that explains what the editors found interesting or important about the work.

**Decision letter after peer review:**

Thank you for submitting your article "Intracellular growth of *Chlamydia trachomatis* leads to global histone hypermethylation by impairing demethylation" for consideration by *eLife*. Your article has been reviewed by 3 peer reviewers, and the evaluation has been overseen by a Reviewing Editor and Dominique Soldati-Favre as the Senior Editor.

The reviewers have discussed their reviews with one another, and the Reviewing Editor has drafted this to help you prepare a revised submission.

*Essential Revisions:*

This valuable study by Charendoff et al. provides interesting observations related to global histone hypermethylation in host cells, during *Chlamydia trachomatis* infections. The core observation they report is that the host histones are highly hypermethylated during infection, and this appears to be an amplifying effect due to continuous inhibition of demethylases, in part due to a metabolic shift in the host where succinate amounts (which inhibit demethylases) increases. The data presented are solid, however, additional data and analysis would strengthen the authors conclusions.

1) While the current data are valuable additional studies to address the mechanistic basis for methylation and transcription changes would strengthen the conclusions.

2) The strength of evidence in favor of the author's stated hypotheses varies due to unaddressed methodological hurdles and unclear experimental rationale or data interpretation as described in the reviews.

*Reviewer #1 (Recommendations for the authors):*

A rephrasing of interpretations is needed (cause/consequence issues) is needed. Better understanding of the metabolic impact after infection (if there is altered SAM flux, flux through the TCA cycle etc) would help the manuscript, and the effect of increased succinate (and its connection to demethylase activity) is simplistic.

*Reviewer #2 (Recommendations for the authors):*

1. RNA-seq analysis for Chlamydia infected cells with/without DMKG addition, Figure 6

As a conclusion drawn from the analysis of infected DEGs between the DMKG-treated and untreated populations, the authors state that "histone hypermethylation does not globally enhance nor repress the host response to infection."(line 265-266). However, the result section also states that "About one third of those (364) were no longer differentially expressed when DMKG was added to the culture medium, indicating that increasing intracellular levels of αKG during infection impacts the host cell's transcriptional response to infection (Figure 6B)."(line 238-240). Given these observations, it would be helpful to clarify how the conclusion that histone hypermethylation does not globally enhance or repress the host response to infection can be reconciled with the finding that a substantial subset of infection-induced DEGs is sensitive to DMKG treatment. In this context, further examination of the transcriptional regulatory regions of these genes-particularly by assessing the correlation between changes in H3K4me3 levels and gene expression for genes that are upregulated upon infection but reverted by DMKG-would strengthen the interpretation.

2. Chlamydia infected cell images for histone methylation Figure 1A, B

Showing only immunofluorescence images of infected cells makes it difficult to assess how histone modification states in host cells change before and after infection. Although bar graphs comparing histone modification levels between uninfected and infected cells are provided, immunofluorescence images of uninfected cells should also be included. In addition, higher-magnification images of infected cells would be helpful for better visualization. The same concern applies to Figure 1B.

3. Figure 6E

Regarding the interpretation of Figure 6E, it should be noted that the change of SAM level also effects on m6A methylation (m6A58) on tRNA which plays a role for tRNA stability, and this possibility should be discussed.

*Reviewer #3 (Recommendations for the authors):*

Please find below several specific comments regarding experimental rationale, data interpretation, and grammar in the manuscript.

A revised manuscript would be greatly improved by (i) the addition of experiments which more directly rule out the role of histone methylases; (ii) performing the demethylase cofactor treatment in a demethylase mutant cell line/in the presence of a dominant negative demethylase/in the presence of the JIB-04 inhibitor to assess the specificity of the effect; and by (iii) addressing the caveat that many assays (specifically, biochemical and genome-wide -omics experiments) include both cells which exhibit histone hypermethylation and those that do not, based on the heterogeneity observed in the immunofluorescent assays.

Line 24 – suggested edit: "Chromatin dynamics are…"

Line 40-41 – suggested edit: I would recommend rewriting this sentence because it gives the impression that Chlamydia does not synthesize any metabolites required for growth and instead relies entirely on the host to obtain all metabolites. (One could argue that the latter is true for any intracellular pathogen that obtains, e.g., carbon and nitrogen sources from the host, regardless of their biosynthetic capacity, thereby making this a moot point for Chlamydia. In other words, the author's statement needs clarification.)

Figure 1, Figure S1A – comment: Because the authors do not include images of an uninfected control in Figure 1, I suggest explicitly stating that the comparison of infected to uninfected cells (i.e., the plots in the lower part of Figure 1A and the images in Figure S1A) reveals that the increase in hypermethylation is specific to the infected condition. It may also be convenient to separate out the results in Figure 1A into separate panels to make referencing these results more convenient.

Figure 1, Figure S1A – comment: It seems necessary for the authors to comment on the heterogeneity of hypermethylation observed by IFA. For example, the authors report that only 10-20% of nuclei are hypermethylated in immortalized cells (though I assume this includes both infected and uninfected cells in the field – nevertheless, it seems likely to be below 50% of infected cells), whereas it appears that basically every infected cell has a brightly stained nuclei in the organoid infected with *C. trachomatis* (and I assume here that *C. trachomatis* does not grow as readily as it does in immortalized cells, raising a question as to what extent bacterial growth is the key determinant here) but the staining is very weak in cells infected with C. muridarum in the organoid, despite similar behavior to *C. trachomatis* in immortalized cells.

Line 88-89 – comment: If I am interpreting this sentence correctly, the authors indicate that they only detect one of the examined histone modifications in their proteomic analysis. I would suggest that the authors state in this passage that a possible reason for this is because the modifications are not present in every infected cell (i.e., the results presented in Figure 1), which would reasonably reduce the chance of detecting all modifications. I am also curious as to why H3K79 does not appear in the plot in Figure 1C (I assume this is not analogous to H3C1 K 80?).

Line 113-114 – comment: It would be helpful if the authors stated what they are comparing ciprofloxacin to, e.g., what antibiotic would have been suitable if not for its interference with host mitochondrial function and why is mitochondrial function so important here? I assume the authors intend to make a metabolic argument, but it would be good to spell this out.

Line 111-112 – comment: The authors could state more explicitly their hypothesis here before the sentence "If true…". That is, stating that they are testing whether increased bacterial replication leads to increased histone methylation. Relatedly, I wonder if the authors tested the hypothesis that bacterial protein synthesis is required for the observed phenotype. Could a short treatment of chloramphenicol ahead of the 48 h timepoint block histone hypermethylation?

Figure 3B – comment: The interpretation of these data is complicated by the fact that treatment with JIB-04 does not appear to increase histone methylation in uninfected cells and that treatment begins from a point at which histone methylation is already increased (i.e., the infected condition). In fact, it appears that the 1 µM treatment actually decreases H4K20me3 fluorescence intensity in uninfected cells. It seems it would be worth testing a broader range of concentrations or conditions to establish that the inhibitor works as expected in the uninfected control. Relatedly, that infection potentiates the effect of inhibiting histone demethylases appears to suggest the opposite of the stated hypothesis. That is, if the concentration of inhibitor is actually sufficient to inhibit demethylases in both infected and uninfected cells, then the most parsimonious explanation for the drastically increased amount of signal in infected cells treated with inhibitor would be that there is more active methylation under the untreated condition, not less demethylation.

Figure 3C – comment: I find this experiment difficult to rationalize. Would one not expect overexpression of a specific demethylase to reduce the amount of cognate methylation regardless of whether that methylation arose due to increased methylation or decreased demethylation? I find the direct measurement of demethylase activity in Figure 3D to be the most compelling evidence in favor of the author's hypothesis in this section of results, but I think that these data would be greatly enhanced by including analogous experiments that test the activity of methylases. The measurement of SAM amounts is consistent with decreased methylation, but this raises the question of how much population-level methylation activity is required to increase histone methylation late in infection, especially given the heterogeneous behavior reported in Figure 1 (that is, could reduce SAM amounts not arise from infected cells which do not exhibit hypermethylation, which appear to be the majority?). Even in individual cells in which SAM amounts are supposedly lower, is it not conceivable that the rate of methylation or the overall amount of methylation could still be dictated by methylase activity precisely because there is less demethylase activity? In other words, wouldn't lower demethylase activity enhance the ability of a lowered rate of methylation to have a meaningful effect?

Results section – "Histone hypermethylation is the result of reduced demethylase activity late in infection" – suggestion: This section of text is a single ~40-line block. I would recommend breaking it up into multiple paragraphs. This comment applies to various sections of the text.

Line 172-175 – comment: is there a literature reference to support this claim? I agree with the rationale I am just curious.

Figure 4B-C – comment: Why is a representative image not provided for the media refreshment condition in Figure 4B?

Figure S2 – suggestion: The authors should adjust the y-axis so that it does not cut off at 108 IFU/mL which gives a misleading impression of the effect size of treatment. I also question the validity of the statistics performed in Figure S2F (and presumably in all of the IFU experiments in this figure), where the data points in the two experimental groups almost entirely overlap, yet a p-value of 0.0011 is reported. The authors should double check this. The authors report using a ratio-paired t-test, but this seems like a test most suitable for a before/after experiment where the samples are actually paired, i.e. derived from the same experimental subject or biologically matched sample; it is not clear how pairing would be accomplished in the experiment as designed.

Line 192-193 – comment: I assume the authors mean "the effect of the combination [on methylation] was not tested," but it is unclear because the preceding passages refer to IFU assays where the authors did test the combination of DMKG and iron treatment (Figure S2F).

Line 197 – suggestion: "imbalance" rather than "unbalance"

Figure 5 – comment: The claim that methylation is happening "outside" H3K4me3-enriched chromatin areas suggests that there should be new, infection-specific peaks, or at least regions of increased read density, and presumably requires stronger supporting evidence than the marginal increase in genome-wide read coverage shown in Figure 5C (that is, shouldn't there be regions of the chromosome outside of peaks with measurably increased read density that are specific to infected samples? Do such regions hint at biological implications? Etc.). The authors note that the number of peaks between infected and uninfected samples are the same, which suggests that there is not a global increase in binding of methylated histones to DNA. It is hard to rationalize from these data why the authors observe cells in which immunostaining of methylated histones is greatly increased. I wonder whether the results would be different if a sorting had been applied prior to the immunoprecipitation, so as to enrich for those cells with high levels of methylated histones. As is, I think the authors need to state the caveat that these data include the many infected cells which do not exhibit increased histone methylation, at least by IFA, and that the interpretation of the data must be carried out accordingly.

Figure 3C – suggestion: The authors might consider converting these data into a heatmap to make them more legible. The plots are very small and it is a lot of information that I think would be enhanced by reorganization. At the very least both of the plots could be in a log scale to make the comparison easier. As an aside, did this analysis include a control gene which the authors did not expect to respond to infection and/or DMKG? It's not obvious to me but should be included.

Line 251 – edit: "…boost or repress…" use of "boost" here feels imprecise

Figure 6E – comment: The results with tRNA abundance are very interesting. For the assay of individual tRNA amounts, the comparison to the DMKG-treated infected sample would be helpful to establish that this behavior correlates with histone hypermethylation. I recognize this is reported for the RNA-seq data, but it raises the question of why this condition was not included when these confirmatory experiments were performed.

Line 332-334 – comment: I think the authors should reflect on this passage again; they measured demethylase activity for one histone modification in samples comparable to those in which they (i) did not robustly detect histone methylation at the proteomic level (e.g., Figure 1C) and (ii) for which they ostensibly observe only a mild increase in DNA bound to methylated histones (Figure 5). The data in Figure 3D are also normalized so as to obscure the variance in the uninfected control, which likely accentuates the measured effect from this assay. To argue from the results of this single demethylase activity experiment that cells in which there is no other strong evidence of hypermethylation are actually experiencing hypermethylation seems questionable, especially because a loss of demethylase activity could easily be accompanied by a loss in methylase activity (as indicated by the reduced amounts of SAM) and therefore a net zero change in overall methylation.

Lines 358-366 – comment: The argument the authors are making here is not entirely clear to me but seems pivotal to their rationale. I would suggest trying to revise this passage so as to emphasize the key points more clearly. For example, the authors highlight the significance of differences in an enzyme's affinity for cofactors, etc., but do not give an example of how this would explain their results. In addition, they do not explain the significance of increased GADD45A and GADD45B expression – what are these proteins? What do they do?

General comment: The authors should review the manuscript for trivial typos or grammatical errors; it would be tedious to point them out one by one – some are indicated above.

[Editors' note: further revisions were suggested prior to acceptance, as described below.]

Thank you for resubmitting your work entitled "Intracellular growth of *Chlamydia trachomatis* leads to global histone hypermethylation by impairing demethylation" for further consideration by *eLife*. Your revised article has been evaluated by Dominique Soldati-Favre (Senior Editor), a Reviewing Editor, and two of the original reviewers.

The manuscript has been improved but there are some remaining issues that you might want to address (e.g., with clarifications in the text), as outlined below:

*Reviewer #1 (Recommendations for the authors):*

I think the authors have done a very careful job in their revision, and the conclusions and interpretations are better supported by the data now. This is a very interesting paper, which I strongly support for final publication.

*Reviewer #3 (Recommendations for the authors):*

This revised manuscript makes several important improvements over the previous version that both enhance clarity and help define the mechanistic basis. Crucially, the FRET-based SAM measurements are a helpful indication that altered metabolism of SAM is not likely to explain hypermethylation. Additional clarification and qualification of the methods and analyses help to establish the rigor of the argument being made in the manuscript. I am providing the following comments to clarify some points I raised in the original evaluation of the manuscript which may be useful for the authors to consider more thoroughly.

(1) In response to my concern about the JIB-04 treatment condition, the authors raise an important point about possible buffering of the compound's potency in the presence of competitive inhibitor iron and/or aKG. However, I do not think that this addresses the main issue, which is the question of whether the inhibitor work as expected in the authors' hands. In other words, buffering or not, if the inhibitor works, some concentration of JIB-04 should increase methylation in uninfected cells. Validating this aspect of the experiment would only strengthen the authors argument that infected cells are X-fold more sensitive to JIB-04. Moreover, the buffering argument raises the critical possibility that, if the inhibitor is working as expected, and if a metabolic/nutritional basis explains the results, then the effect of the inhibitor should be readily titrated away by the addition of iron and/or DMKG (perhaps more readily in infected vs uninfected cells). Such an experiment would provide strong evidence that demethylases specifically are responsible for the reported behaviors and is related to the following comment.

(2) The authors respond to my suggestion to demonstrate the specificity of cofactor treatment with several arguments that I am not sure address the core issue I was trying to raise. To reiterate: my suggestion was to use, e.g., dominant-negative demethylase expression, to demonstrate that the altered methylation under cofactor treatment was acting via demethylases. Because iron and a-KG are pleiotropic agents, this strikes me as an important experiment. The authors suggest the dominant negative effect of their constructs is not strong, but this would be expected under a condition where demethylase activity is already low (note that this is an interpretation of the data in favor of the authors' central argument) and the point here would be to observe a lack of response to cofactor treatment. The same logic would apply to cofactor treatment in the presence of JIB-04 inhibitor. By measuring a specific methylation mark in the presence or absence of both cofactor and dominant negative demethylase (or inhibitor), one would expect to see that the cofactor treatment would only alter methylation prevalence in the absence of the dominant negative allele (if the model is correct). The authors suggest that DMKG supplementation in the presence of JIB-04 would be difficult to interpret because both treatments slow bacterial growth (the data suggest this is a very mild growth defect), but why not use iron, which is a/the strict competitor for JIB-04 binding and which has the same effect as DMKG on methylation? Ultimately, I agree with the authors that the data demonstrating sensitivity of infected cells to JIB-04 and to DMKG and iron agree with one another and point towards a culprit (demethylases) but the suggested experiment would help establish that link causally.

(3) Regarding the rationale for overexpressing demethylases, the authors note (in response to Reviewer #2) that "we included this experiment to show that increasing the expression of one demethylase only restored demethylation of its cognate target." I would like to note that this disagrees with the rationale provided in the corresponding Results section which states: "If [the hypothesis that lysine demethylation by JmjC demethylases is low late in infection] is correct, overexpression of a given JmjC demethylase should reverse the enhanced methylation of its target residue." This is relevant to the concern I raised about this experiment, which I am not sure is fully addressed by the authors' response. Specifically, I was concerned with the notion that this experiment addresses the hypothesis that is stated in the manuscript, because demethylase overexpression should reduce cognate methylation no matter the source of methylation. If, by contrast, the rationale for this experiment is to show that hypermethylation is reversible when enough demethylase activity is present, then I have no major issue with the experiment. But I would emphasize that I think this is a very different interpretation from the one offered in the Results section which implies that this experiment addresses whether hypermethylation is caused by low demethylase activity late in infection. (Note that this interpretation is explicitly stated again in the same paragraph of the Results section after the data are described.) It also appears to be at odds with the notion that there is a metabolic/cofactor limitation on demethylase activity if simply expressing more demethylase (which requires cofactor) is sufficient to reverse hypermethylation. Is demethylase gene expression reduced during infection?

(4) With respect to the point raised about Figure 6C, I would like to clarify that while I may be mistaken about whether the data are in logarithmic scale, clearly the data are computing two different kinds of fold change, one that produces negative fold-changes (the microarray data – negative fold-change being usually accomplished by log transformation), and one that does not (the RT-qPCR data, which is reported as values >0). Why not be consistent here for the sake of comparison and legibility of the figure? I did not raise an issue about microarray versus RT-qPCR, I raised an issue about being able to easily interpret the data. Of course, this was simply a suggestion, and the authors may do as they wish. Finally, I raised a point about a control gene. By this I did not mean a normalization gene (which does not guarantee that its expression is unchanged unless validated empirically!) but rather a gene which the microarray data suggests is not changed by treatment and/or infection which can be confirmed by RT-qPCR identically to the other genes in this figure (i.e., using ACTN as normalization). This is typically a good internal control for any such analysis specifically because changes in RT-qPCR data can arise due to changes in the expression of a normalization gene and other artifacts.

# Reply: Author response

**DOI:** 10.7554/eLife.110111.sa2

Essential Revisions:

Reviewer #1 (Recommendations for the authors):

A rephrasing of interpretations is needed (cause/consequence issues) is needed. Better understanding of the metabolic impact after infection (if there is altered SAM flux, flux through the TCA cycle etc) would help the manuscript, and the effect of increased succinate (and its connection to demethylase activity) is simplistic.

We thank this reviewer for insightful comments which help us clarify several aspects of the manuscript. Based on this recommendation, we performed novel experiments and rephrased some of the conclusions as detailed above.

Reviewer #2 (Recommendations for the authors):

1. RNA-seq analysis for Chlamydia infected cells with/without DMKG addition, Figure 6

As a conclusion drawn from the analysis of infected DEGs between the DMKG-treated and untreated populations, the authors state that "histone hypermethylation does not globally enhance nor repress the host response to infection."(line 265-266). However, the result section also states that "About one third of those (364) were no longer differentially expressed when DMKG was added to the culture medium, indicating that increasing intracellular levels of αKG during infection impacts the host cell's transcriptional response to infection (Figure 6B)."(line 238-240). Given these observations, it would be helpful to clarify how the conclusion that histone hypermethylation does not globally enhance or repress the host response to infection can be reconciled with the finding that a substantial subset of infection-induced DEGs is sensitive to DMKG treatment. In this context, further examination of the transcriptional regulatory regions of these genes-particularly by assessing the correlation between changes in H3K4me3 levels and gene expression for genes that are upregulated upon infection but reverted by DMKG-would strengthen the interpretation.

We apologize for the confusion our poor wording has created. In this part of the manuscript we explain in detailed that the transcriptional shift due to histone hypermethylation is not unidirectional, as there are as many DEGs which display up-regulation of transcription as DEGs displaying the reverse effect (down-regulation). We have deleted the sentence “histone hypermethylation does not globally enhance nor repress the host response to infection.” which caused the confusion.

The suggestion to focus on H3K4me3 presents the risk of sending a contradictory message: we insist on the fact that each gene integrates remodelling of the entire methylation landscape. Therefore, focusing on H3K4me alone, in the absence of information on other potential marks, could be misleading and we decided against this suggestion.

2. Chlamydia infected cell images for histone methylation Figure 1A, B

Showing only immunofluorescence images of infected cells makes it difficult to assess how histone modification states in host cells change before and after infection. Although bar graphs comparing histone modification levels between uninfected and infected cells are provided, immunofluorescence images of uninfected cells should also be included. In addition, higher-magnification images of infected cells would be helpful for better visualization. The same concern applies to Figure 1B.

Images of non-infected cells are provided in Figure S1. Regarding the ideal magnification for Figure 1, we privileged the importance to show fields containing several infected and several non-infected cells, for the reader to be able to appreciate that hypermethylation only occurs in a subset of infected cells.

3. Figure 6E

Regarding the interpretation of Figure 6E, it should be noted that the change of SAM level also effects on m6A methylation (m6A58) on tRNA which plays a role for tRNA stability, and this possibility should be discussed.

We had pointed to this possibility in the discussion of the initial submission (l. 470). Since our new data suggest that SAM levels might be largely stable, we changed our comment to the following: “Finally, several non-coding RNA were differentially regulated during infection, in a DMKG sensitive manner, including several tRNAs. Modification in the activity of Fe(II)/aKG-dependent RNA demethylases might be implicated in this phenomenon as methylations are among the post-transcriptional modifications regulating tRNA stability (Adami and Bottai, 2020).”

Reviewer #3 (Recommendations for the authors):

Please find below several specific comments regarding experimental rationale, data interpretation, and grammar in the manuscript.

A revised manuscript would be greatly improved by (i) the addition of experiments which more directly rule out the role of histone methylases; (ii) performing the demethylase cofactor treatment in a demethylase mutant cell line/in the presence of a dominant negative demethylase/in the presence of the JIB-04 inhibitor to assess the specificity of the effect; and by (iii) addressing the caveat that many assays (specifically, biochemical and genome-wide -omics experiments) include both cells which exhibit histone hypermethylation and those that do not, based on the heterogeneity observed in the immunofluorescent assays.

We thank this reviewer for her/his important comments. Reponses to these three points are as follows:

(i) We also considered the possibility that infected cells displayed increased histone methyl transferase (HMT) activity. SAM is notoriously labile. For this reason, in vitro assays to measure HMT require to add exogenous SAM to cell extracts to detect any HMT activity, which would not allow us to test activity based on endogenous SAM levels.

Instead, we used a ratiometric sensor to measure SAM concentration in cells (PMID 34937909). We observed cell-to-cell heterogeneity in SAM levels, as previously reported. Thus, in some cells, SAM might actually be limiting, which could explain why only a fraction of cells display histone hypermethylation. Chlamydia inclusions develop asynchronously, which allows to observe, 40 hpi, a continuum of early (low bacterial load) to late (high bacterial load) stages of infection. There was no correlation between bacterial load and SAM level, and this level was globally similar when comparing infected and non-infected cells. This experiment supports our hypothesis that protein hypermethylation is not due to an increase in SAM during infection. Still, we cannot rule out the possibility that increase in SAM availability late in the infectious cycle contributes to protein hypermethylation. This is discussed in a new paragraph in the Discussion section.

(ii) Overexpression of catalytic dead version of KDMs do not have a strong dominant negative effect in our experimental set up (for instance, overexpression of dKDM4A slightly increases H3K9me3 levels compared to overexpression of dKDM5A as a negative control but the difference is not significant, Figure 3C). As for using a demethylase mutant line, the multiplicity of genes coding for one demethylase activity makes this approach difficult.

Supplementation of DMKG in the presence of JIB-04 is doable but since each of this chemical slows down bacterial replication, the interpretation of the data would be difficult.

(iii) Indeed, several of our assays did not offer single cell resolution. The new data using the ratiometric sensor emphasize the cell-to-cell heterogeneity in metabolite levels such as SAM level and we hope that new methods will become available to repeat some of our observations at single cell resolution.

Line 24 – suggested edit: "Chromatin dynamics are…"

Done, thank you

Line 40-41 – suggested edit: I would recommend rewriting this sentence because it gives the impression that Chlamydia does not synthesize any metabolites required for growth and instead relies entirely on the host to obtain all metabolites. (One could argue that the latter is true for any intracellular pathogen that obtains, e.g., carbon and nitrogen sources from the host, regardless of their biosynthetic capacity, thereby making this a moot point for Chlamydia. In other words, the author's statement needs clarification.)

Done, thank you

Figure 1, Figure S1A – comment: Because the authors do not include images of an uninfected control in Figure 1, I suggest explicitly stating that the comparison of infected to uninfected cells (i.e., the plots in the lower part of Figure 1A and the images in Figure S1A) reveals that the increase in hypermethylation is specific to the infected condition. It may also be convenient to separate out the results in Figure 1A into separate panels to make referencing these results more convenient.

We clarified this point. The quantifications were done on infected and non-infected cells of the same infected coverslip, as stated in the text (l. 68) and in the figure legend. As suggested, we separated Figure 1A in two panels.

Figure 1, Figure S1A – comment: It seems necessary for the authors to comment on the heterogeneity of hypermethylation observed by IFA. For example, the authors report that only 10-20% of nuclei are hypermethylated in immortalized cells (though I assume this includes both infected and uninfected cells in the field – nevertheless, it seems likely to be below 50% of infected cells), whereas it appears that basically every infected cell has a brightly stained nuclei in the organoid infected with *C. trachomatis* (and I assume here that *C. trachomatis* does not grow as readily as it does in immortalized cells, raising a question as to what extent bacterial growth is the key determinant here) but the staining is very weak in cells infected with *C. muridarum* in the organoid, despite similar behavior to *C. trachomatis* in immortalized cells.

Indeed, only 10-20% of infected cells displayed histone hypermethylation in HeLa cells. This was quantified by comparing infected versus non-infected cells of the same infected well (the fact that only infected coverslips were imaged and quantified is stated both in the text and in the legend). It is indeed different in the organoid model in which the majority of infected cells show higher H3K9me3 levels than non-infected cells. We now have reproduced this observation in human-derived organoids (not included in this manuscript). This difference can be due to several factors:

- the diffusion of the culture medium might be refrained in matrix-encased organoids, making them more sensitive to scarcity of certain metabolites.

- the composition of culture media is different for the two models.

- SAM levels, which might limit the extent of hypermethylation in HeLa cells, might

not do so in Fallopian tube derived cells.

Finally, note that *C. muridarum* infected organoids were fixed at 30 hpi, because the *C. muridarum* lifecycle is shorter (PMID 33468625). This might explain a lower methylation level compared to organoids infected for 48 hpi by *C. trachomatis*.

Line 88-89 – comment: If I am interpreting this sentence correctly, the authors indicate that they only detect one of the examined histone modifications in their proteomic analysis. I would suggest that the authors state in this passage that a possible reason for this is because the modifications are not present in every infected cell (i.e., the results presented in Figure 1), which would reasonably reduce the chance of detecting all modifications. I am also curious as to why H3K79 does not appear in the plot in Figure 1C (I assume this is not analogous to H3C1 K 80?).

We added a comment l. 92-93 as suggested. H3C1 K80 indeed corresponds to H3K79. H3C1 K80 is the protein sequence in the database, which includes the first translated amino acid (Methionine). The latter is rapidly cleaved and is not included in the traditional numbering of H3 amino acids (same for H4).

Line 113-114 – comment: It would be helpful if the authors stated what they are comparing ciprofloxacin to, e.g., what antibiotic would have been suitable if not for its interference with host mitochondrial function and why is mitochondrial function so important here? I assume the authors intend to make a metabolic argument, but it would be good to spell this out.

We also observed a reduction of histone methylation upon treatment with doxycycline instead of ciprofloxacin. However, the latter affects mitochondrial metabolism, including the TCA cycle. Since a-ketoglutarate and succinate are intermediates of the TCA cycle, doxycycline treatment could affect histone methylation independently of its inhibition of bacterial proliferation. Therefore, we only displayed the data related to ciprofloxacin. We clarified this in the text (l. 119)

Line 111-112 – comment: The authors could state more explicitly their hypothesis here before the sentence "If true…". That is, stating that they are testing whether increased bacterial replication leads to increased histone methylation. Relatedly, I wonder if the authors tested the hypothesis that bacterial protein synthesis is required for the observed phenotype. Could a short treatment of chloramphenicol ahead of the 48 h timepoint block histone hypermethylation?

We changed the text to be more explicit, and we performed additional experiments using chloramphenicol. We observed that chloramphenicol treatment had the same effect as ciprofloxacin, confirming that robust bacterial growth is required for histone hypermethylation to occur. The data have been added in a modified Figure 2B.

Figure 3B – comment: The interpretation of these data is complicated by the fact that treatment with JIB-04 does not appear to increase histone methylation in uninfected cells and that treatment begins from a point at which histone methylation is already increased (i.e., the infected condition). In fact, it appears that the 1 µM treatment actually decreases H4K20me3 fluorescence intensity in uninfected cells. It seems it would be worth testing a broader range of concentrations or conditions to establish that the inhibitor works as expected in the uninfected control. Relatedly, that infection potentiates the effect of inhibiting histone demethylases appears to suggest the opposite of the stated hypothesis. That is, if the concentration of inhibitor is actually sufficient to inhibit demethylases in both infected and uninfected cells, then the most parsimonious explanation for the drastically increased amount of signal in infected cells treated with inhibitor would be that there is more active methylation under the untreated condition, not less demethylation.

We thank the reviewer for careful examination of the data. Indeed, it looked as if 1 μm treatment with JIB-04 decreased H4K20me3 fluorescence. This was due to the fact that in the control well (noted as [JIB-04]=0) we had added the equivalent volume of DMSO (vehicule) as in the [JIB-04]=2 μm well (dilution 1:500 v:v), while in the [JIB-04]=1 μm well DMSO was present at 1:1000. It turns out that 1:500 DMSO slightly increases basal level of H4K20me3 fluorescence. We repeated this series of experiments using stocks of JIB04 at higher concentrations, the new data are presented in New Figure 3B.

Importantly, the observation that the same concentration of JIB-04 leads to an increase of histone methylation in infected cells and not in non-infected cells, is coherent with our data. The inhibitor is taken up similarly by infected and uninfected cells but the potency of the inhibitor will depend partly on levels of iron, aKG and succinate found in the cellular milieu so same concentration of inhibitor may inhibit demethylase activity in cells with higher succinate and/or low aKG and low iron but fail to inhibit demethylase activity in cells with higher iron or aKG or lower succinate. In other words, high iron, high aKG or low succinate will “buffer” JIB-04 and make it less potent since JIB-04 partly acts by competing with the iron (competitively) and the aKG (mixed competitive inhibition) PMID 23792809. The same phenomenon is expected for SD70 and TACH101 that share aspects of the mode of action of JIB-04 regarding partly competing for aKG and/or iron in the catalytic site.

Figure 3C – comment: I find this experiment difficult to rationalize. Would one not expect overexpression of a specific demethylase to reduce the amount of cognate methylation regardless of whether that methylation arose due to increased methylation or decreased demethylation? I find the direct measurement of demethylase activity in Figure 3D to be the most compelling evidence in favor of the author's hypothesis in this section of results, but I think that these data would be greatly enhanced by including analogous experiments that test the activity of methylases. The measurement of SAM amounts is consistent with decreased methylation, but this raises the question of how much population-level methylation activity is required to increase histone methylation late in infection, especially given the heterogeneous behavior reported in Figure 1 (that is, could reduce SAM amounts not arise from infected cells which do not exhibit hypermethylation, which appear to be the majority?). Even in individual cells in which SAM amounts are supposedly lower, is it not conceivable that the rate of methylation or the overall amount of methylation could still be dictated by methylase activity precisely because there is less demethylase activity? In other words, wouldn't lower demethylase activity enhance the ability of a lowered rate of methylation to have a meaningful effect?

The caveat regarding the measure of HMT activity was addressed in (i) page 10. We fully agree that the cell-to-cell heterogeneity in metabolites might account for the observation that only a minority of cells exhibit histone hypermethylation. This hypothesis was strengthened by our observation, during paper revision, that HeLa cells exhibit high cell-to-cell heterogeneity in SAM concentration. Histone hypermethylation might only be possible in cells in which SAM is above a certain threshold. These novel considerations have been added to the discussion in a new paragraph.

Results section – "Histone hypermethylation is the result of reduced demethylase activity late in infection" – suggestion: This section of text is a single ~40-line block. I would recommend breaking it up into multiple paragraphs. This comment applies to various sections of the text.

This section has been divided into 2 sections and the data split into 2 figures.

Line 172-175 – comment: is there a literature reference to support this claim? I agree with the rationale I am just curious.

We are not aware of a discussion of this point in the literature. We routinely perform SDS-PAGE analysis of proteins extracted from infected vs non infected cells (Coomassie blue staining). Bands only detected in infected samples, which correspond almost exclusively to bacterial proteins, represent a significant fraction of the total proteins after 30 h of infection.

Figure 4B-C – comment: Why is a representative image not provided for the media refreshment condition in Figure 4B?

We did not think useful to include the media refreshment condition, as it is very similar to the DMSO condition.

Figure S2 – suggestion: The authors should adjust the y-axis so that it does not cut off at 108 IFU/mL which gives a misleading impression of the effect size of treatment. I also question the validity of the statistics performed in Figure S2F (and presumably in all of the IFU experiments in this figure), where the data points in the two experimental groups almost entirely overlap, yet a p-value of 0.0011 is reported. The authors should double check this. The authors report using a ratio-paired t-test, but this seems like a test most suitable for a before/after experiment where the samples are actually paired, i.e. derived from the same experimental subject or biologically matched sample; it is not clear how pairing would be accomplished in the experiment as designed.

Thanks for pointing this out. We changed the y-axis. We had forgotten to include the statistics for panel S2C, this was added. Regarding panels S2F, we confirm that ratiopaired t-test were applied (progeny can fluctuate from one experiment to the other based on cell batches, the pairing per experiment is recommended). The display of the data has been adapted in the left panel to show the pairing. We kept a histogram display on the right panel for readability.

Line 192-193 – comment: I assume the authors mean "the effect of the combination [on methylation] was not tested," but it is unclear because the preceding passages refer to IFU assays where the authors did test the combination of DMKG and iron treatment (Figure S2F).

This was clarified, thank you

Line 197 – suggestion: "imbalance" rather than "unbalance"

Done.

Figure 5 – comment: The claim that methylation is happening "outside" H3K4me3-enriched chromatin areas suggests that there should be new, infection-specific peaks, or at least regions of increased read density, and presumably requires stronger supporting evidence than the marginal increase in genome-wide read coverage shown in Figure 5C (that is, shouldn't there be regions of the chromosome outside of peaks with measurably increased read density that are specific to infected samples? Do such regions hint at biological implications? Etc.). The authors note that the number of peaks between infected and uninfected samples are the same, which suggests that there is not a global increase in binding of methylated histones to DNA. It is hard to rationalize from these data why the authors observe cells in which immunostaining of methylated histones is greatly increased. I wonder whether the results would be different if a sorting had been applied prior to the immunoprecipitation, so as to enrich for those cells with high levels of methylated histones. As is, I think the authors need to state the caveat that these data include the many infected cells which do not exhibit increased histone methylation, at least by IFA, and that the interpretation of the data must be carried out accordingly.

Indeed, sorting of cells with high bacterial burden would probably have given stronger differences between the two samples. We added two sentences in the result (l. 247) and discussion (l. 406) sections to emphasize this caveat. Still, we think the observations we made are interesting. The appearance of H3K4me3 outside usual peaks suggest that KDMs normally clean these “random” marks.

Figure 3C – suggestion: The authors might consider converting these data into a heatmap to make them more legible. The plots are very small and it is a lot of information that I think would be enhanced by reorganization. At the very least both of the plots could be in a log scale to make the comparison easier. As an aside, did this analysis include a control gene which the authors did not expect to respond to infection and/or DMKG? It's not obvious to me but should be included.

These comments likely refer to Figure 6C. The y axes plot Fold Change (no logarithmic scale in this figure). For several genes we observe a higher range of Fold Change by RTqPCR than by microarray analysis. The observed differences in fold change between microarray and RT-qPCR are well-documented in the literature. Microarrays have a narrower dynamic range and are prone to cross-hybridization, which can lead to underestimation of true fold changes. In contrast, RT-qPCR offers higher sensitivity and specificity, often detecting larger fold changes (PMID: 12000853). Since the experiment aimed at validating the microarray data, we focused on genes showing differential expression. The gene used for normalization (*ACTIN*) was not expected to respond to infection nor DMKG and was indeed stable.

Line 251 – edit: "…boost or repress…" use of "boost" here feels imprecise

“boost” was replaced by “enhance”

Figure 6E – comment: The results with tRNA abundance are very interesting. For the assay of individual tRNA amounts, the comparison to the DMKG-treated infected sample would be helpful to establish that this behavior correlates with histone hypermethylation. I recognize this is reported for the RNA-seq data, but it raises the question of why this condition was not included when these confirmatory experiments were performed.

Having validated the microarray data in Figure 6C we did not consider it necessary to perform northern blot on the DMKG condition. We had instead chosen to measure the effect of JIB-04 on tRNA amount, to test whether it might enhance the effect of infection (the effect was found not significant). We realized later that the data were difficult to interpret as each K_dm_ probably displays different sensitivity to JIB-04 and to DMKG. The data were thus not included in the manuscript.

Line 332-334 – comment: I think the authors should reflect on this passage again; they measured demethylase activity for one histone modification in samples comparable to those in which they (i) did not robustly detect histone methylation at the proteomic level (e.g., Figure 1C) and (ii) for which they ostensibly observe only a mild increase in DNA bound to methylated histones (Figure 5). The data in Figure 3D are also normalized so as to obscure the variance in the uninfected control, which likely accentuates the measured effect from this assay. To argue from the results of this single demethylase activity experiment that cells in which there is no other strong evidence of hypermethylation are actually experiencing hypermethylation seems questionable, especially because a loss of demethylase activity could easily be accompanied by a loss in methylase activity (as indicated by the reduced amounts of SAM) and therefore a net zero change in overall methylation.

Thanks for this remark. Indeed, it is only the decrease of demethylase activity that might be underestimated by looking at the methylation level (possibly indeed because SAM is low). We modified the sentence to clarify this.

Lines 358-366 – comment: The argument the authors are making here is not entirely clear to me but seems pivotal to their rationale. I would suggest trying to revise this passage so as to emphasize the key points more clearly. For example, the authors highlight the significance of differences in an enzyme's affinity for cofactors, etc., but do not give an example of how this would explain their results. In addition, they do not explain the significance of increased GADD45A and GADD45B expression – what are these proteins? What do they do?

The text was modified for clarification.

General comment: The authors should review the manuscript for trivial typos or grammatical errors; it would be tedious to point them out one by one – some are indicated above.

Thanks, this has been done.

[Editors’ note: what follows is the authors’ response to the second round of review.]

The manuscript has been improved but there are some remaining issues that you might want to address (e.g., with clarifications in the text), as outlined below:

Reviewer #1 (Recommendations for the authors):

I think the authors have done a very careful job in their revision, and the conclusions and interpretations are better supported by the data now. This is a very interesting paper, which I strongly support for final publication.

Thank you for this comment and for taking the time to examine our revised version.

Reviewer #3 (Recommendations for the authors):

This revised manuscript makes several important improvements over the previous version that both enhance clarity and help define the mechanistic basis. Crucially, the FRET-based SAM measurements are a helpful indication that altered metabolism of SAM is not likely to explain hypermethylation. Additional clarification and qualification of the methods and analyses help to establish the rigor of the argument being made in the manuscript. I am providing the following comments to clarify some points I raised in the original evaluation of the manuscript which may be useful for the authors to consider more thoroughly.

(1) In response to my concern about the JIB-04 treatment condition, the authors raise an important point about possible buffering of the compound's potency in the presence of competitive inhibitor iron and/or aKG. However, I do not think that this addresses the main issue, which is the question of whether the inhibitor work as expected in the authors' hands. In other words, buffering or not, if the inhibitor works, some concentration of JIB-04 should increase methylation in uninfected cells. Validating this aspect of the experiment would only strengthen the authors argument that infected cells are X-fold more sensitive to JIB-04. Moreover, the buffering argument raises the critical possibility that, if the inhibitor is working as expected, and if a metabolic/nutritional basis explains the results, then the effect of the inhibitor should be readily titrated away by the addition of iron and/or DMKG (perhaps more readily in infected vs uninfected cells). Such an experiment would provide strong evidence that demethylases specifically are responsible for the reported behaviors and is related to the following comment.

The ability of JIB-04 to increase histone methylation even in non-infected cells is clear in the data reporting fluorescence intensities (dot plots). However, the effect is seen on a minority of cells, thus more than 5 experiments would be required to reach significance in the histograms. Since the effect of JIB-04 on non-infected cells was more obvious for H3K9me3 than H4K20me3, we now show the H3K9me3 data in the main figure, and the H4K20me3 data in the associated supplementary panel.

As suggested, since we previously showed JIB-04 was a competitor for iron (PMID 23792809), we now tested whether its effect would be dampened by the addition of iron. This was indeed the case, and the new data are shown in Figure 5_ figure supplement 2.

(2) The authors respond to my suggestion to demonstrate the specificity of cofactor treatment with several arguments that I am not sure address the core issue I was trying to raise. To reiterate: my suggestion was to use, e.g., dominant-negative demethylase expression, to demonstrate that the altered methylation under cofactor treatment was acting via demethylases. Because iron and a-KG are pleiotropic agents, this strikes me as an important experiment. The authors suggest the dominant negative effect of their constructs is not strong, but this would be expected under a condition where demethylase activity is already low (note that this is an interpretation of the data in favor of the authors' central argument) and the point here would be to observe a lack of response to cofactor treatment. The same logic would apply to cofactor treatment in the presence of JIB-04 inhibitor. By measuring a specific methylation mark in the presence or absence of both cofactor and dominant negative demethylase (or inhibitor), one would expect to see that the cofactor treatment would only alter methylation prevalence in the absence of the dominant negative allele (if the model is correct). The authors suggest that DMKG supplementation in the presence of JIB-04 would be difficult to interpret because both treatments slow bacterial growth (the data suggest this is a very mild growth defect), but why not use iron, which is a/the strict competitor for JIB-04 binding and which has the same effect as DMKG on methylation? Ultimately, I agree with the authors that the data demonstrating sensitivity of infected cells to JIB-04 and to DMKG and iron agree with one another and point towards a culprit (demethylases) but the suggested experiment would help establish that link causally.

The data now presented in Figure 5_ figure supplement 2 show that the ability of iron supplementation to prevent hypermethylation is reduced when JIB-04 is also added.

As stated by Reviewer #3, this experiment strengthens our conclusions and we are thankful for suggesting it.

(3) Regarding the rationale for overexpressing demethylases, the authors note (in response to Reviewer #2) that "we included this experiment to show that increasing the expression of one demethylase only restored demethylation of its cognate target." I would like to note that this disagrees with the rationale provided in the corresponding Results section which states: "If [the hypothesis that lysine demethylation by JmjC demethylases is low late in infection] is correct, overexpression of a given JmjC demethylase should reverse the enhanced methylation of its target residue." This is relevant to the concern I raised about this experiment, which I am not sure is fully addressed by the authors' response. Specifically, I was concerned with the notion that this experiment addresses the hypothesis that is stated in the manuscript, because demethylase overexpression should reduce cognate methylation no matter the source of methylation. If, by contrast, the rationale for this experiment is to show that hypermethylation is reversible when enough demethylase activity is present, then I have no major issue with the experiment. But I would emphasize that I think this is a very different interpretation from the one offered in the Results section which implies that this experiment addresses whether hypermethylation is caused by low demethylase activity late in infection. (Note that this interpretation is explicitly stated again in the same paragraph of the Results section after the data are described.) It also appears to be at odds with the notion that there is a metabolic/cofactor limitation on demethylase activity if simply expressing more demethylase (which requires cofactor) is sufficient to reverse hypermethylation. Is demethylase gene expression reduced during infection?

Thank you for re-expressing this point. We modified the result and Discussion sections accordingly. To answer your last question “Is demethylase gene expression reduced during infection?”: The microarray data showed that some demethylases are up-regulated in infection, some are down-regulated. KDM5A is down (- 1.3 fold) and KDM5C is up (+ 1.3 fold). KDM4A and KDM4B are down (-1.4 and -1.5 respectively) and KDM4E is up (+ 1.2). KDM6B is up (+ 1.5 fold) and KDM7A is up (+2.5 fold).

(4) With respect to the point raised about Figure 6C, I would like to clarify that while I may be mistaken about whether the data are in logarithmic scale, clearly the data are computing two different kinds of fold change, one that produces negative fold-changes (the microarray data – negative fold-change being usually accomplished by log transformation), and one that does not (the RT-qPCR data, which is reported as values >0). Why not be consistent here for the sake of comparison and legibility of the figure? I did not raise an issue about microarray versus RT-qPCR, I raised an issue about being able to easily interpret the data. Of course, this was simply a suggestion, and the authors may do as they wish. Finally, I raised a point about a control gene. By this I did not mean a normalization gene (which does not guarantee that its expression is unchanged unless validated empirically!) but rather a gene which the microarray data suggests is not changed by treatment and/or infection which can be confirmed by RT-qPCR identically to the other genes in this figure (i.e., using ACTN as normalization). This is typically a good internal control for any such analysis specifically because changes in RT-qPCR data can arise due to changes in the expression of a normalization gene and other artifacts.

Thank you for the clarification. With all due respect, we decided to keep the figure as it is for the following reason:

1. Regarding the representation of the data. Indeed, we could have chosen to convert the micro-array data to be consistent with the RT-qPCR data, which are classically represented with values >0. However, we made the choice to keep the representation of the microarray data exactly as they appear in Table S5, where the data are reported with some negative fold changes, so that the reader can directly see how these graphs were generated.

2. Regarding the usefulness of adding a gene that is not changed by DMKG treatment and/or infection. The purpose of the RT-qPCR experiments was to validate the micro-array data. We selected the 10 genes in an non-biased way by the 10 genes that were most responsive to DMKG addition and infection. It turned out that they included genes that were up-regulated by infection, and genes that were down-regulated. In addition, some of these 10 genes showed opposite response to DMKG addition. If the changes were due to changes in the expression of the normalization gene, we would expect all genes to suffer the same bias and be shifted in the same way. We believe that this non-biased focus on 10 genes reach the goal of the validation process and that repeating these experiments to add a focus on a stable transcript is not needed.
